# Chronic wound microenvironment mediates selection of biofilm-forming multi drug resistant *Staphylococcus epidermidis* with capability to impair healing

**DOI:** 10.21203/rs.3.rs-2562300/v1

**Published:** 2023-02-17

**Authors:** Miroslav Dinic, Rebecca Verpile, Jingjing Meng, Jelena Marjanovic, Jamie L Burgess, Lisa Plano, Suzanne Hower, Seth R Thaller, Santanu Banerjee, Hadar Lev-Tov, Marjana Tomic-Canic, Irena Pastar

**Affiliations:** 1 Wound Healing and Regenerative Medicine Research Program, Dr. Phillip Frost Department of Dermatology and Cutaneous Surgery, University of Miami Miller School of Medicine, Miami, FL, USA; 2 Group for Probiotics and Microbiota-Host Interaction, Laboratory for Molecular Microbiology, Institute of Molecular Genetics and Genetic Engineering, University of Belgrade, Belgrade, Serbia; 3 Department of Microbiology and Immunology, University of Miami Miller School of Medicine, Miami, FL, USA; 4 Department of Surgery, University of Miami Miller School of Medicine, Miami, FL, USA

**Keywords:** *Staphylococcus epidermidis*, chronic wounds, antimicrobial resistance, biofilm, wound healing

## Abstract

Venous leg ulcers (VLU) are the most common chronic wounds characterized by bacterial biofilms and perturbed microbiome. *Staphylococcus epidermidis* is primarily known as skin commensal beneficial for the host, however, some strains can form biofilms and cause infections. By employing shotgun metagenomic sequencing we show that genetic signatures of antimicrobial resistance, adhesion and biofilm formation in VLU isolates correlate with *in vitro* bacterial traits. We demonstrate that the capability of chronic wound isolates to form biofilms and elicit IL-8 and IL-1β expression in human *ex vivo* wounds, correlates with the non-healing outcomes in patients with VLU. In contrast, commensal strains were incapable of surviving in the human *ex vivo* wounds. We show that major fitness traits of *S. epidermis* from VLU involve genes for resistance to methicillin and mupirocin, while the biofilm formation relied on the minimal number of genetic elements responsible for bacterial binding to fibronectin and fibrinogen. This underscores the importance of the emergence of treatment resistant virulent lineages in patients with non-healing wounds.

## Introduction

Venous leg ulcers (VLUs) represent the most common chronic wound affecting millions of patients and leading to billions of dollars of associated healthcare costs annually^1,2^. The chronic ulcer microenvironment is a suitable site for the development of biofilms that can compromise healing and contribute to severe complications^3,4^. While the role of *Staphylococcus aureus* in chronic wound pathology has been extensively studied^4–6^, the contribution of its close relative, *Staphylococcus epidermidis*, to impaired healing has not yet been evaluated.

As one of the first commensals, *S. epidermidis* contributes to healthy cutaneous barrier by inhibiting colonization of pathogens and regulating the immune response^7^. *S. epidermidis* also has an educating role for the variety of resident immune cells, priming them to respond to their pathogenic relatives like *S. aureus*^8,9^. *S. epidermidis* can also stimulate keratinocytes to produce broad spectrum antimicrobial peptides (AMP)^10^. Alongside its beneficial role, *S. epidermidis* could carry a reservoir of antimicrobial resistance (ABR) genes and virulence factors which places this microbe to the list of “accidental” pathogens known to cause nosocomial and device-associated infections^11–13^. *S. epidermidis* can carry genes encoding polysaccharide intercellular adhesins (*icaADBC)* and accumulation-associated protein (*aap)* linked to biofilm formation, but also fibronectin (*embp*), fibrinogen (*sdrG*) and keratin/collagen I (*sdrF*) binding genes important for adhesion to different surfaces^11^. Biofilms and disturbed microbiota are also a hallmark of nonhealing chronic wounds including VLUs^3,14^. While comparative whole-genome sequencing (WGS) methods have been utilized for determining pathogenicity among disease associated *S. epidermidis* strains^12^, chronic wound isolates have not been analyzed before.

## Results and Discussion

**Figure 1.**
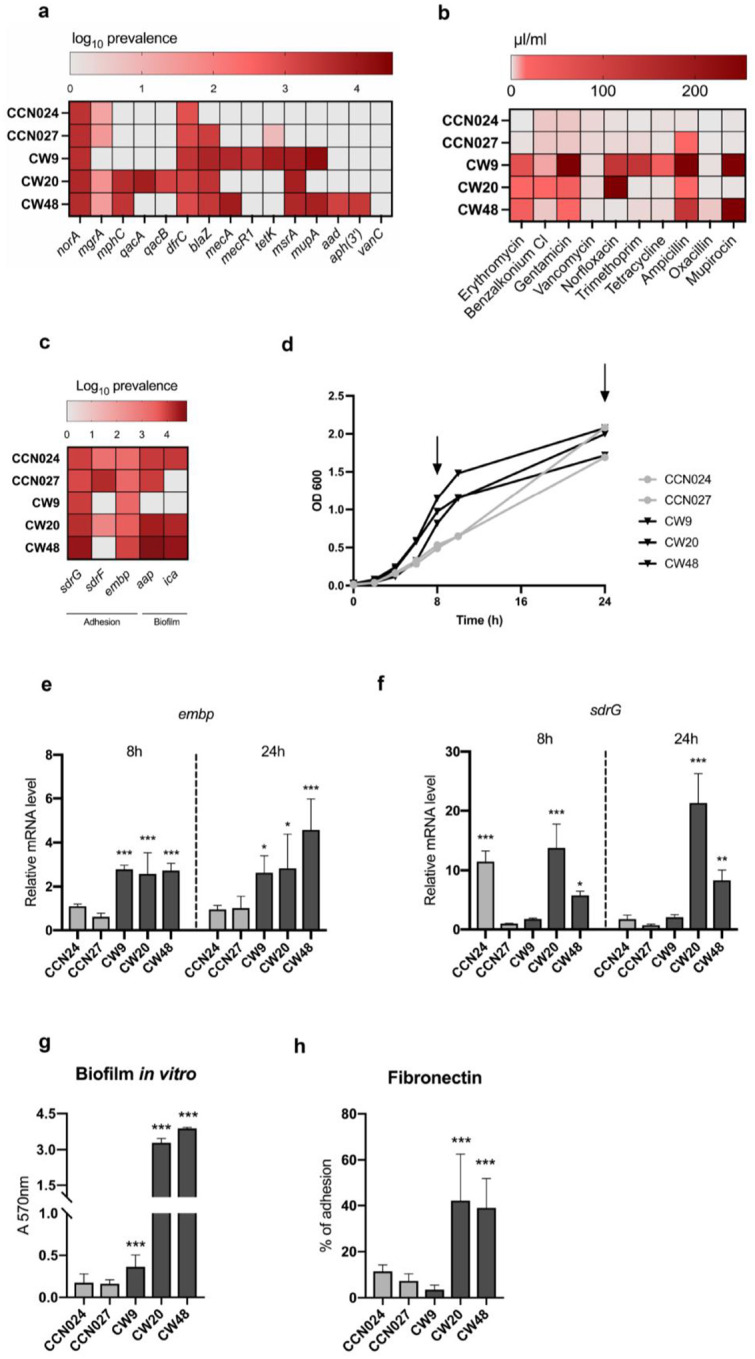
*S. epidermidis* isolates from VLUs show resistance to broad-spectrum antibiotics and higher capacity to form biofilm and bind to fibronectin, *in vitro*. (**a**) Heat-map representing the antibiotic resistance gene (ABR) prevalence in healthy skin and chronic wound isolates followed by (**b**) MIC50 values of selected antibiotics showing susceptibility of *S. epidermidis* isolates from both origins. Data are presented from 3 independent experiments with 3 technical replicates. (**c**) Heat-map showing prevalence of known *S. epidermidis* adhesion and biofilm associated genes across all isolates. (**d**) The growth curves of *S. epidermidis* isolates showing better fitness of chronic wound isolates with the arrows pointing to exponential (8h) and stationary phases (24h) when the bacteria were collected for gene expression analysis. (**e**, **f**) Gene expression of the fibronectin-binding (*embp*) and the fibrinogen-binding (*sdrG*) genes through growth phases in all isolates assessed by qRT-qPCR; data is presented as mean ± SD from results obtained from three independent experiments (n=3–6). (**g**) Cristal violet assay showing biofilm forming potential of all isolates after 72h of incubation. (**h**) Percentage (%) of adhesion of all isolates to fibronectin; data are presented as the mean ± SD from results obtained from three independent experiments (n=6–12). One-way ANOVA followed by Dunnett’s *post hoc* test was used to compare the results of all isolates relative to CCN027 healthy skin strain (*p<0.05, **p<0.01, ***p<0.001).

We report the first metagenomic and functional characterization of *S. epidermidis* isolates from chronic VLU (CW9, CW20 and CW48) and comparison to commensal skin strains (CCN024 and CCN027). Using the WGS we identified prevalence of *ABR* in VLU strains, not present in commensal isolates, confirmed in MIC assays (Fig. 1a&b). Specifically, *mupA* and *mecA* genes and associated resistance to mupirocin and oxacillin/methicillin, respectively, were key features of the VLU isolates CW9 and CW48. In contrast to CW9 and CW48, strain CW20 showed susceptibility to oxacillin and mupirocin. *MecA* has been associated with *S. epidermidis* virulence in systemic and device-related infections12, however mupirocin resistance in pathogenic isolates has not been reported before. In addition, higher MIC values for benzalkonium chloride, an antiseptic used in chronic wound management, were detected in VLU isolates but not in commensals, while only CW20 strain harbored the qacA/qacB encoding resistance to quaternary ammonium compounds (Fig. 1a&b). Our findings raise significant concern for VLU patients, as both mupirocin and benzalkonium chloride are often used for pre-operative skin decolonization of *S. aureus*^15^ but may result in selection of multi-drug resistant (MDR) S*. epidermidis*.

We further evaluated the gene repertoire encoding the components required for binding to host extracellular matrix (ECM) and detected most of these genes in all isolates (Fig. 1c). The expression of *embp* and *sdrG*, present in all strains, was evaluated during the exponential and stationary growth (Fig. 1d), revealing significantly higher *embp* expression in VLU in comparison to healthy skin isolates regardless of the growth phase (Fig. 1e). Expression of *sdrG* was upregulated in two chronic wound isolates, CW20 and CW48, while in CW9 strain remained comparable to levels in the commensals (Fig 1f). We also detected higher expression of *sdrG* in commensal CCN024, not sustained at the stationary phase. Altogether, the differences in *embp* and *sdrG* expression indicated better colonizing and biofilm forming properties of VLU isolates and correlated with potential of CW20 and CW48 to form biofilm *in vitro*, while CW9 showed higher biofilm production in comparison to skin commensals (Fig. 1g). Moreover, in accordance with *embp* expression, CW20 and CW48 strains showed a significantly high level of binding to human fibronectin (Fig. 1h), whereas all strains showed low ability to attach to collagen (**Suppl. Fig 1**).

**Figure 2.**
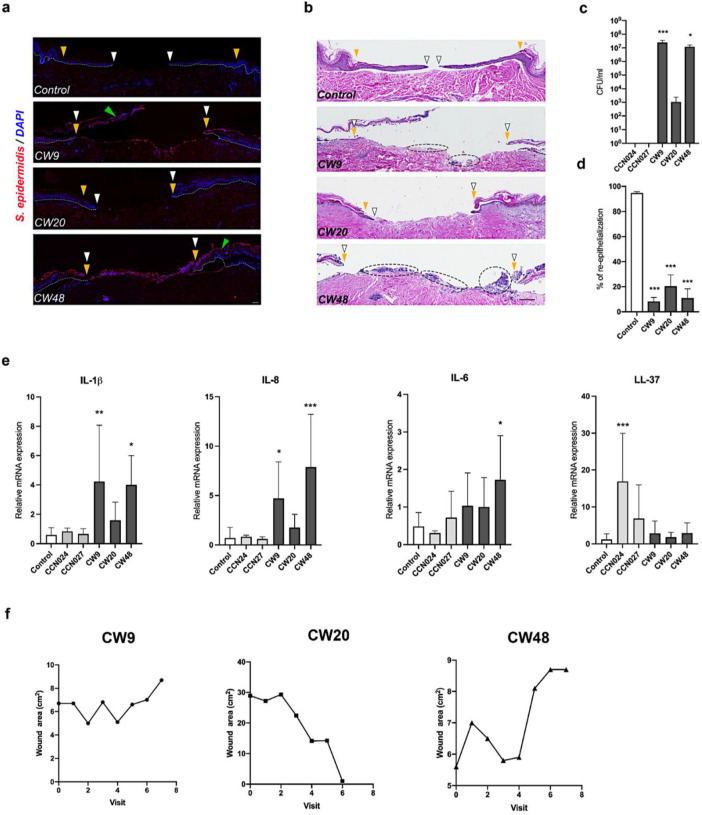
The ability of chronic wound *S. epidermidis* isolates to form pro-inflammatory biofilm in *ex vivo* human wounds associates with clinical outcomes in patients. (**a**) Immunostaining of *S. epidermidis* biofilm on *ex vivo* human wounds day 4 post-wounding; control – no infection. Yellow arrows showing wound edge, white arrows indicate migrating epithelium visualized by DAPI staining of keratinocyte nuclei, with epidermis separated from dermis caused by VLU isolates (green arrows) (**b**) H&E staining of uninfected wound (control), and wounds infected with CW9, CW20, and CW48 showing wound edge (yellow arrows) and epithelial tongue (white arrows) on day 4 post-wounding. Bacterial aggregates stained by hematoxylin are indicated by dashed lines; scale bar = 200 μm. (**c**) Bacterial growth in *ex vivo* wounds on day 4. (**d**) Percentage of re-epithelization of *ex vivo* wounds non-infected and infected with VLU isolates; data is presented as mean ± SD from three independent experiments (n=3). One-way ANOVA followed by Dunnett’s *post hoc* test was used to compare the results of all isolates relative to uninfected control or CCN027 (*p<0.05, ***p<0.001). (**e**) Gene expression analysis of pro-inflammatory cytokines and antimicrobial peptides; data is presented as the mean ± SD from three independent experiments (n=3–8). One-way ANOVA followed by Dunnett’s *post hoc* test was used to compare the gene expression levels relative to untreated control (*p<0.05, **p<0.01, ***p<0.001). (**e**) Clinical healing trajectories of VLU study subjects correlate with the *ex vivo* biofilm formation of corresponding *S. epidermidis* isolates. Ulcer size of VLU patients was assessed weekly during 8 weeks of standard of care.

To assess if *S. epidermidis* isolates contribute to high non-healing rates of VLUs observed in patients, we used a human *ex vivo* wound model and evaluated biofilm formation and wound closure upon infection. Immunostaining of *S. epidermidis* and H&E in wounds infected with CW9 and CW48 showed localization of bacteria in the wound bed, overlying the epidermis, and between the epidermal-dermal junction at the wound edges (Fig. 2a&b). We also detected high bacterial load for CW9 and CW48, while the strain CW20 had limited growth in human *ex vivo* wounds (Fig. 2a–c), despite its potency to form biofilm *in vitro*. In contrast to VLU isolates, healthy skin isolates were unable to grow in the *ex vivo* wounds (Fig. 2c). The potent biofilm-forming ability of CW9 and CW48 indicated correlation of their virulence primarily with the strains’ ABR, rather than the mere presence of biofilm-associated genes. In addition, CW9 formed a wound biofilm even in the absence of *ica* operon, *aap* and s*drF*, thought to be indispensable for biofilm formation^11^. Next, we evaluated re-epithelialization in *ex vivo* wounds 4 days post-infection. While control uninfected wounds showed high levels of wound closure, VLU isolates inhibited healing (Fig. 2b & d) and caused detachment of the epidermis from the dermis which can be associated with Staphylococci proteolytic activity^16^. However, genes encoding cysteine (*ecp*) and serine (*esp*) exoproteases were detected among all isolates (**Suppl Fig. 2**). We also analyzed the presence of genes encoding the *agr* system, a virulence factor regulator^17^, and the cassette chromosome recombinase-encoding genes *ccrA* and *ccrB*^18^ and found no association with strains’ biofilm formation (**Suppl Fig. 3&4**). Next, we examined gene expression of the pro-inflammatory markers and AMPs. Only biofilm formed by CW9 and CW48 elicited a significant induction of IL-1β and IL-8 (Fig. 2e). Infection with CW48 also resulted in increased expression of IL-6, while commensal strain CCN024 was the only inducer of AMP LL-37 (Fig. 2e). Importantly, the ability of VLU isolates to form the biofilm in *ex vivo* wounds correlated with the healing outcomes in patients with VLU (Fig. 2f). CW9 and CW48 strains were isolated from VLUs with no improvement in healing during 8 weeks of standard of care, which correlates with the biofilm forming ability in the *ex vivo* wounds (Fig. 2f). In contrast, CW20 isolate which showed low levels of survival in *ex vivo* wound*s*, and lack of *mupA* and *mecA*, was isolated from an ulcer that healed (Fig. 2f).

Our study revealed the major fitness traits of *S. epidermidis* isolates from chronic VLU involving *ABR*, specifically *mecA* and *mupA*, while the biofilm formation relied solely on *embp* and *SdrG*. Furthermore, we show that the capability of chronic wound isolates to form biofilms and elicit pro-inflammatory response in human wound model, correlates with the non-healing outcomes in patients with VLU. We also recognize limitations of our study, including a limited number of VLU and commensal strains. Future studies will involve characterization of the strains from a larger number of patients and evaluations of polymicrobial biofilms. Regardless, our data underscores concern of multi-drug resistant (MDR) *S. epidermidis* in chronic wounds suggesting increased infection risk elsewhere in the body of affected patients. Increasing bacterial resistance among chronic wound isolates and the association with impaired healing necessitates development of novel antimicrobial and antibiofilm approaches aimed to prevent selection of MDR strains.

## Materials and Methods

### Patient population

Patients presenting to the wound clinic at the University of Miami with chronic VLUs were recruited after informed consent was obtained under approved IRB protocol (#20180468). Participants presented with non-infected target ulcers >2 cm^2^ and at least one month duration. Exclusion criteria included use of systemic or topical antibiotics, immunosuppressants, and cellular therapy in the month preceding the study. All subjects received the standard-of-care including a dressing regimen and a 4 layered compression bandage system. Patients were monitored in the wound clinic for 8 weeks or until wound closure was achieved. Tissue that was debrided was collected at the initial visit for bacterial isolation and characterization.

### Bacterial strains and growth conditions

Chronic wound isolates of *S. epidermidis* (CW9, CW20, CW48 strains) were isolated from debridement VLU tissue using a standard microbiology approach^1^. Human commensal *S. epidermidis* CCN024, and CCN027 strains, were isolated from healthy volunteers and characterized by phenotypic and qPCR identification techniques as previously described^1,2^. All bacteria were preserved with 20% glycerol at −80° C until the time of culture in Tryptic Soy Broth (TSB) at 37°C, overnight.

### DNA isolation and shotgun sequencing

DNA from *S. epidermidis* isolates was extracted using the DNeasy Blood and Tissue Kit (Qiagen) with a modified lysis step. Enzymatic lysis was performed by using lysostaphin (0.5 mg/ml) in 20 mM Tris EDTA buffer supplemented with 1.2% Triton X-100 for 1 hour at 37°C. Preparation and sequencing of DNA libraries were carried out in the John P. Hussman Institute for Human Genomics Center for Genome Technology. Each sample was sequenced at 5 million paired-end 100 base reads on the Illumina NextSeq500. Sequencing data analysis was performed using Partek Flow genomic analysis software (Partek Inc. (2020). Partek^®^ Flow^®^ (Version 10.0) [Computer software]. https://www.partek.com/partek-flow/). Reads were aligned to the reference libraries using STAR-2.7.8a, filtered to remove the duplicates, and quantified using the Partek Expectation/Maximization (E/M) algorithm. Antibiotic resistance gene prediction was conducted using the Comprehensive Antibiotic Resistance Database (CARD).3 The data are available under BioProject ID PRJNA906272.

### Statistical analysis

All data are presented as mean values ± standard deviation (SD). One-way ANOVA followed by Dunnett’s *post hoc* test were used to compare all the isolates relative to selected healthy skin isolate or uninfected control. A *p* value less than 0.05 was considered statistically significant. The statistical analysis was performed and graphs were prepared using GraphPad Prism 8 software.

Additional methods are provided in *Supplementary data*.

## Data Availability

The authors declare that all data supporting the findings of this study are available within the article and its supplementary information files or from the corresponding author upon reasonable request. Raw data and analyzed metagenomic data supporting the findings in this study have been deposited under BioProject ID PRJNA906272.
